# Posterior epidural migration of lumbar disc fragment: Case reports and literature review

**DOI:** 10.1097/MD.0000000000028146

**Published:** 2021-12-10

**Authors:** Youngmin Oh, Jongpil Eun

**Affiliations:** Department of Neurosurgery, Biomedical Research Institute, Jeonbuk National University Medical School and Hospital, Jeonju, Korea.

**Keywords:** back pain, cauda equine syndrome, herniated lumbar disc, posterior epidural mass, radiculopathy, sequestration

## Abstract

**Introduction:**

Posterior epidural migration of lumbar disc fragment (PEMLDF) is a very rare condition that may lead to a serious neurological deficit such as cauda equina syndrome. Magnetic resonance imaging (MRI) findings can often result in cases of PEMLDF being misdiagnosed as extradural masses of other origin or epidural hematomas. In this study, we reported four additional cases of PEMLDF and reviewed the relevant literature.

**Patient concerns:**

We present four patients with PEMLDF. The mean age of the patients was 53.5 years. Two patients suffered from cauda equine syndrome, and the other two patients complained of radiculopathy.

**Diagnosis:**

The MRI findings in each case showed masses with slightly high signal intensity in T2-weighted images, as well as heterogenous and peripheral rim enhancement after contrast enhancement. In some patients there was a tract-like enhancement extending from the outer aspect of the disc to the posterolateral epidural space. A definitive diagnosis was made intraoperatively.

**Interventions:**

We performed laminectomy and discectomy in all patients.

**Outcomes:**

The PEMLDF patients with radiculopathy had no complaints of weakness or pain after surgery. Both patients with cauda equine syndrome showed a total recovery post-surgery.

**Conclusions:**

Early diagnosis and treatment via laminectomy and discectomy is critical to achieving the best postoperative outcomes. Understanding the patient's history, recognizing the similar signal intensity of the mass and intervertebral disc on MRI scans, and looking for peripheral rim enhancement, are the keys to the correct diagnosis of PEMLDF.

## 1 Introduction

Intervertebral disc sequestration, in which an extruded disc migrates within the spinal canal, is responsible for up to 28.6% of all disc herniations.^[1,2]^ Caudal and paracentral displacements are the most common patterns, with posterior epidural migration of the lumbar disc fragment (PEMLDF) being very rare. Since Lombardi reported the first posterior epidural migration of a sequestrated lumbar intervertebral disc fragment case in 1973 approximately 120 cases have been reported in the literature^.^^[1,3–6]^ The rarity of the condition has meant that the underlying cause of posterior disc fragment migration is not well understood. Moreover, while PEMLDF may lead to serious neurological deficit, the condition is difficult to diagnose and to treat. In this study, we report four additional cases of PEMLDF and reviewed the relevant literature. This case report has been approved by institutional review board of Jeonbuk National University Hospital, who waived the need for obtaining informed consent, because this study was retrospective.

## 2 Case report

### 2.1 Case 1

**Figure 1:**
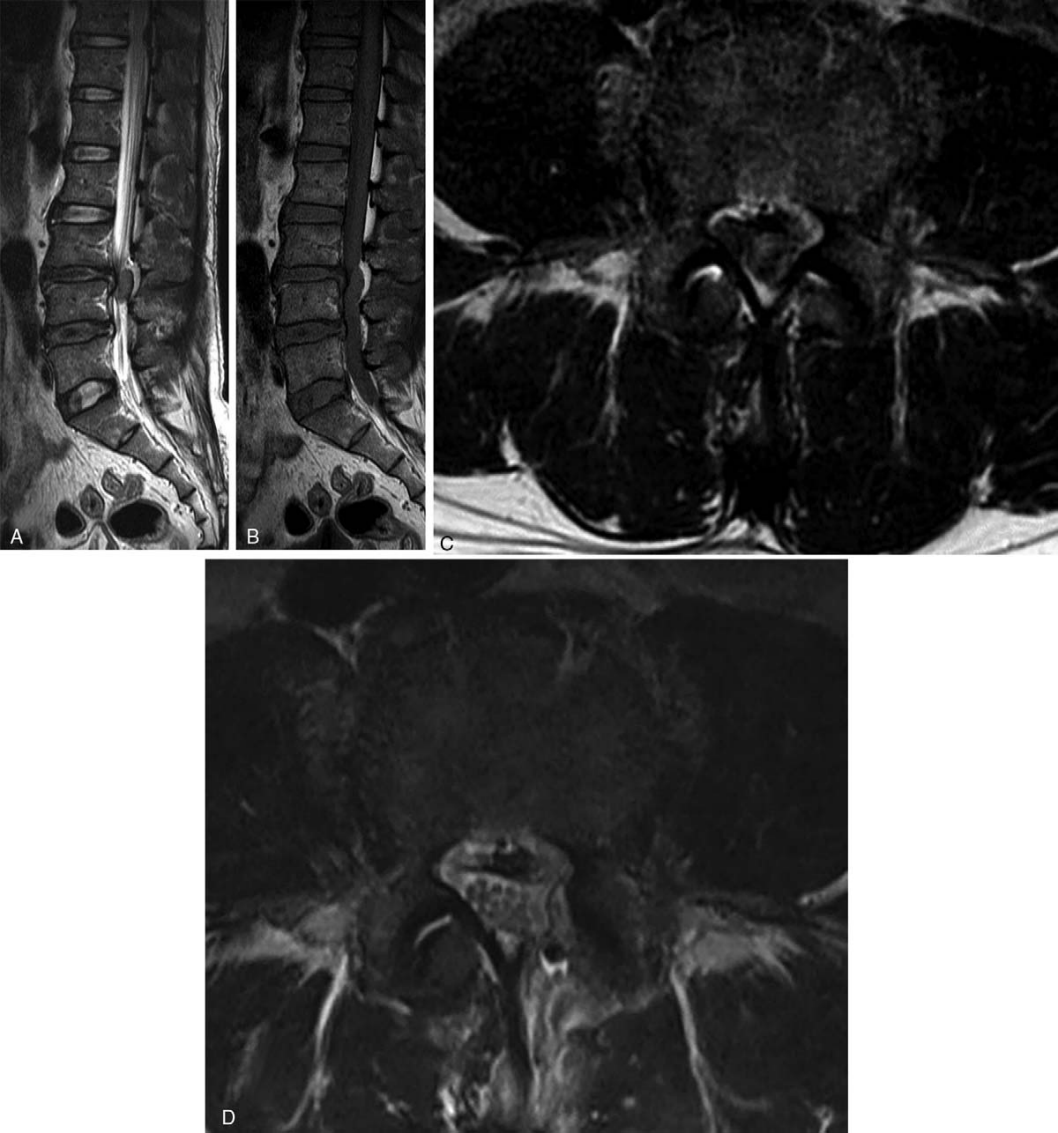
Preoperative sagittal T2-weighted magnetic resonance imaging (MRI) (A), sagittal T1-weighted MRI (B), and axial T2-weighted MRI (C) of the lumbar spine demonstrated an extradural mass lesion in the left-lateral and posterior epidural space at the level of L3-L4 with severe thecal sac compression (white arrow). Postoperative axial T2-weighted MRI (D) showed laminectomy and no thecal sac compression.

**Figure 2:**
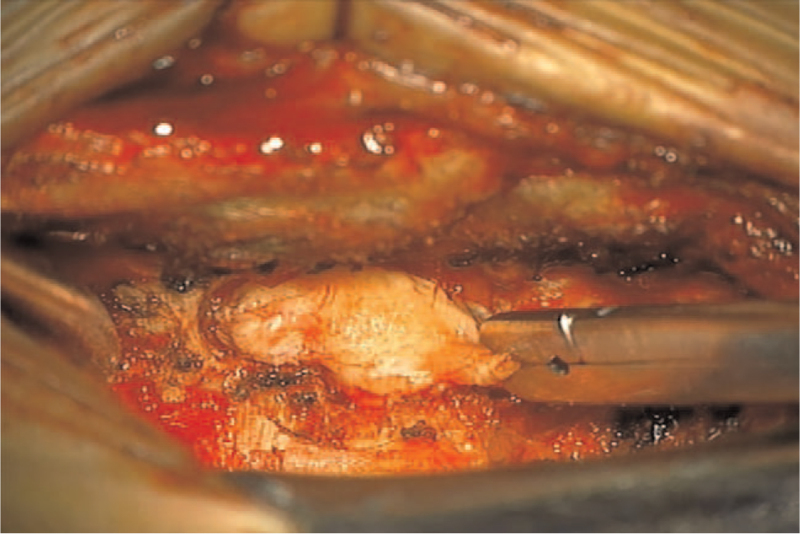
Intraoperative finding at L3-L4 level following a laminectomy showing a very large dorsally migrated disc fragment.

A 45-year-old male presented with 1 week of progressive back pain radiating to his left leg and an acute onset of bilateral leg weakness. He had no history of trauma or surgery and no other medical illness. On the neurological examination, he was unable to dorsiflex his ankles and great toes against gravity (1/5 weakness of bilateral ankle & great toe dorsiflexion). He also complained of voiding difficulty. Magnetic resonance imaging (MRI) of the lumbar spine indicated a somewhat heterogenous and T2-weighted hyperintense mass lesion in the left lateral and posterior epidural space at L3–4 (Fig. 1). Significant disc degenerations at L3-4 and L4-5 disc space accompanied this. Due to the acute onset of symptoms, as well as the severity of neurologic involvement, the patient was taken to surgery for exploration and removal of the epidural mass. An L3-4 left hemi-laminectomy was performed, revealing a very large dorsally migrated disc fragment that erupted as soon as the ligamentum flavum was removed (Fig. 2). The large mass, which was displacing the thecal sac to the right, was removed in several large pieces, tracking down to the L3-4 disc space until all neurologic elements were satisfactorily decompressed. At that time, the annular tear was visualized on the left side of the L3-4 disc space; the fragment was sent to pathology and confirmed to be disc material. Confident that the extruded disc material was completely removed, the wound was irrigated and closed. Postoperatively, his leg pain and numbness resolved immediately. By the six-week follow-up appointment, he had regained full strength in his ankles, and reported no bowel, bladder, or sexual dysfunction.

### 2.2 Case 2

**Figure 3:**
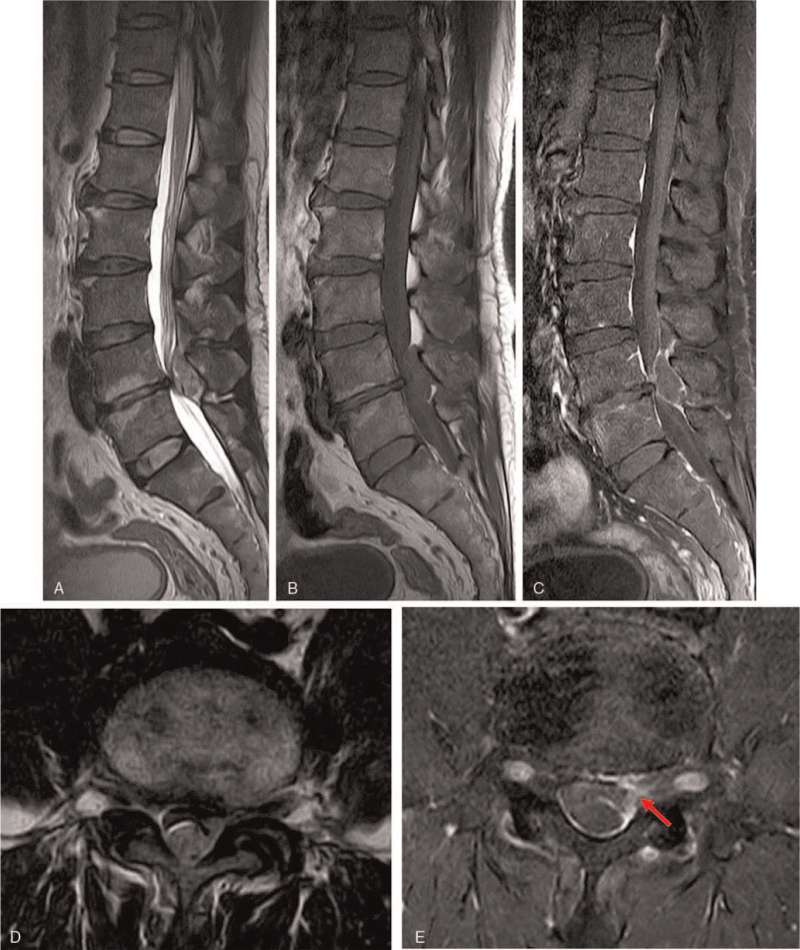
Preoperative MRI of the lumbar spine without and with contrast enhancement. Sagittal (A) and axial (D) T2-weighted MRI and sagittal T1-weighted MRI (B) showed a posterior epidural mass. Enhanced T1-weighted MRI in the sagittal and axial planes showed a lesion with heterogenous ring-like enhancement at the L4-L5 level and demonstrated a sequestrated fragment with posterior migration (C and E). On the axial images, the route of migration in the form of a tract-like enhancement extending from the outer aspect of the disc to the posterior epidural space was found (Red arrow).

A 46-yeal-old male presented with lower back pain and left leg weakness that had lasted for 1 week. On examination, he presented with left ankle dorsiflexion weakness (2/5), left lower extremity numbness, urinary incontinence and abnormal gait. The MRI demonstrated a posterior epidural mass lesion at the L4-5 level and an endplate degeneration adjacent to the L4-5 disc space (Fig. 3). This mass showed peripheral rim enhancement, and on the axial images a route of migration in the form of a tract-like enhancement extending from the outer aspect of the disc to the posterior epidural space (Fig. 3). An L4-5 left hemi-laminectomy was performed, and it was confirmed that this was an extruded disc. Histopathology was also consistent with a disc herniation. At his 2-year follow-up the patient had no residual weakness or left lower extremity numbness.

### 2.3 Case 3

**Figure 4:**
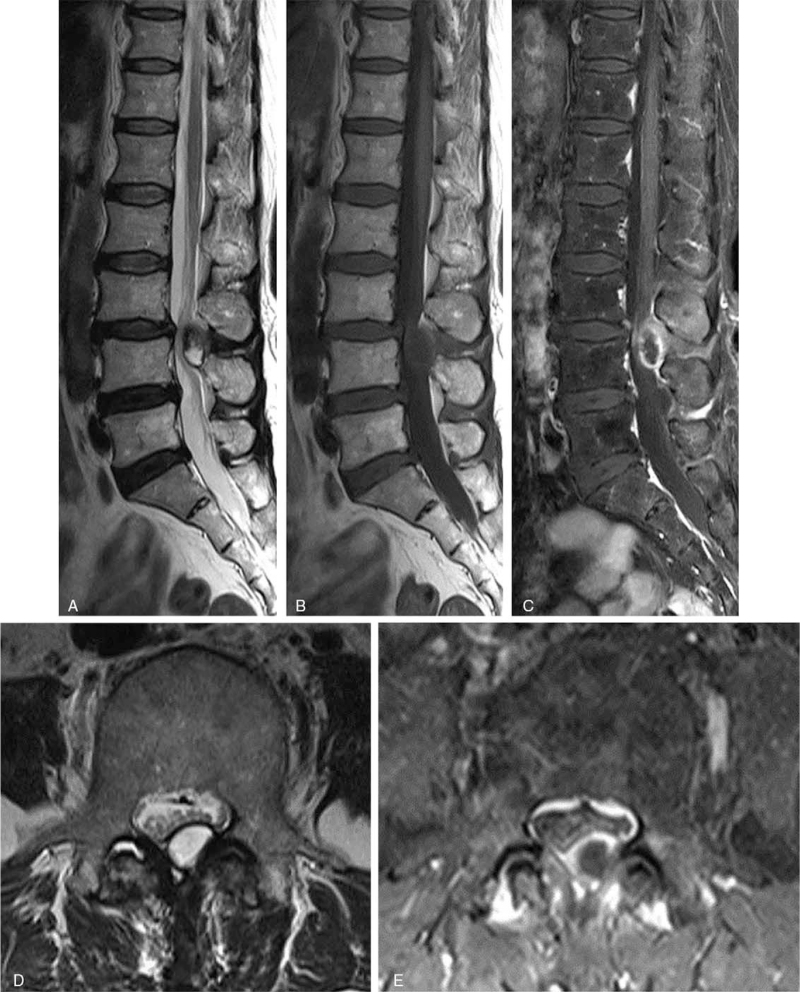
Preoperative lumbar MRI without contrast enhancement demonstrated a posterior epidural lesion at the L3-4 level with thecal sac compression (A, B, and D). After contrast enhancement, this mass-like lesion showed heterogenous signal intensity and peripheral rim enhancement (C and E).

A 67-year-old female presented with radiating pain throughout her bilateral lower extremities that had lasted for 1 month. She showed no leg weakness. A lumbar MRI demonstrated a posterior epidural lesion at the L3-4 level with thecal sac compression (Fig. 4). This mass-like lesion showed heterogenous signal intensity and peripheral rim enhancement. Based on these radiological findings, a differential diagnosis of cyst tumor (e.g., schwannoma or disc sequestration) was made. An L3-4 laminectomy revealed a dark extradural lesion compressing the thecal sac, which histopathology confirmed to be sequestrated disc material. Within 3 days postoperatively her condition had markedly improved, and at a meeting 3 months post-surgery she had no complaints.

### 2.4 Case 4

**Figure 5:**
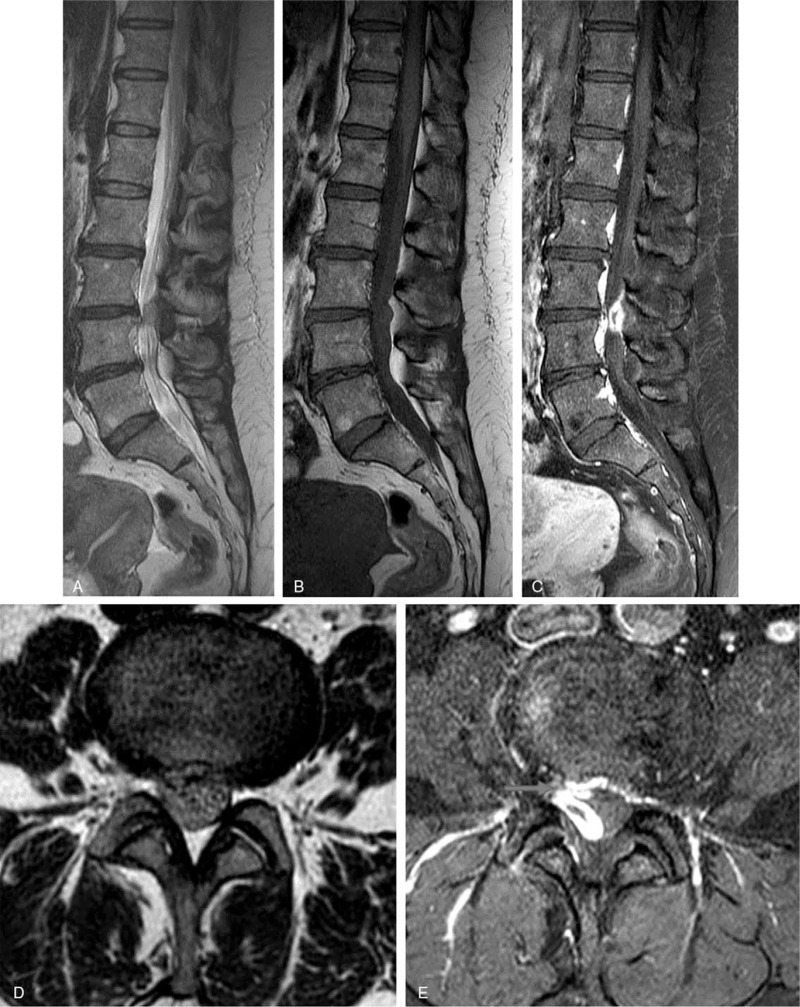
Preoperative MRI of the lumbar spine without and with contrast enhancement. Sagittal T2-weighted MRI (A) and sagittal (B) and axial T1-weighted MRI (D) showed a posterolateral epidural mass compressing the thecal sac. Enhanced T1-weighted MRI in the sagittal and axial planes showed a lesion with heterogenous ring-like enhancement at the L3-L4 level and demonstrated a sequestrated fragment with posterior migration (C and E). On the axial images, the route of migration in the form of a tract-like enhancement extending from the outer aspect of the disc to the posterolateral epidural space was found (Red arrow).

A 53-year-old female presented with lower back pain and bilateral leg radiating pain that had lasted for 2 months. She had no history of trauma. On exam, she had bilateral ankle dorsiflexion weakness (4/5). The MRI revealed a 1 cm X 2 cm sized posterior epidural mass lesion at the L3-4 level (Fig. 5). This lesion showed peripheral rim enhancement and also track-like enhancement on axial images (Fig. 5D). An L3-4 left hemi-laminectomy was performed, which confirmed this mass to be sequestrated disc material. Her symptoms improved markedly in the immediate aftermath of the operation.

## 3 Discussion

Only 120 cases of PEMLDF, including the present cases, are reported in the English-language literature. PEMLDF occurs infrequently because it requires passing through anatomical barriers such as the posterior longitudinal ligament, the peridural and lateral membrane, the epidural venous plexus, epidural fat, the nerve root, and the dura.^[7,8]^ PEMLDF occurs mostly in middle-aged persons (mean age 53.11 years), possibly owing to the dynamics of spinal degeneration associated with aging.^[5,9]^ Most commonly (40% of reported cases), posterior migration occurs at L3-L4, possibly due to the anatomical relationship between the nerve root and disc space in the horizontal plane, which appears to compromise the barrier role of the nerve root at this level.^[4,9]^ In our case series, three among the four patients (75%) showed posterior migration at L3-L4.

Clinically, PEMLDF can present with symptoms as diverse as lower back pain, radiculopathy, or cauda equina syndrome (CES).^[10]^ CES is clinically rare in disc herniation cases, occurring in 1.8 per million persons within the general population. Notably, the incidence of CES is higher in PEMLDF cases.^[11]^ According to Elsharkawy et al approximately 50% of PEMLDF patients presented with CES and neurologic deficits.^[4]^ As an explanation, they suggested that the small size of the posterior epidural space is conducive to neural structure compression, increasing the chances of neurologic deficits. Among our case series, two patients (50%) presented with CES (Table 1).

**Table 1:** Clinical features of our case series with radiological findings.

No	Age	Sex	Symptoms	Signs	Level	Surgery	MRI contrast enhancement	Outcome Follow up status
1	45	M	CES (1 d)	Ankle dorsiflexion G1/G1	L3–L4	Laminectomy & discectomy	Not available	Able to walk without cane
				Voiding difficulty				No bladder incontinence
2	46	M	CES (7 d)	Ankle dorsiflexion G3/G2	L4–L5	Laminectomy & discectomy	Rim enhancement	Able to walk without cane
				Voiding difficulty				No bladder incontinence
3	69	F	Radiculopathy	Ankle dorsiflexion G4/G4	L3–L4	Laminectomy & discectomy	Rim enhancement	Improved power, no pain
4	54	F	Radiculopathy	Ankle dorsiflexion G4/G4	L3–L4	Laminectomy & discectomy	Rim enhancement	Improved power, no pain

The gold standard for diagnosis is a gadolinium enhanced lumbar spine MRI scan.^[12,13]^ In the vast majority of cases (up to 80%), the sequestrated fragments exhibit high signal intensity on T2-weighted images relative to the degenerated disc of origin and low signal intensity on T1-weighted images.^[13]^ Inflammatory changes in the local environment cause an increase in fluid content in the extruded material, which is responsible for T2 hyperintensity.^[8]^ Chen et al suggested that high signal intensity on T2-weighted images could be explained as the result of herniated material possessing a higher water content than an intact disc.^[13]^ Contrast agent must be always used in order to rule out the most common topographical differential diagnosis. Most disc fragments show peripheral contrast enhancement, which may be attributed to an inflammatory response with granulation tissue and newly formed vessels around the sequestrated tissue,^[13]^ as shown in this report.

MRI findings can be confounded by atypical images mimicking an epidural tumor, abscess, hematoma, and facet cyst, leading to diagnostic difficulty and uncertainly in management. Thus, clinicians should remain vigilant for the differential diagnosis of other diseases. Tumors usually enhance uniformly on gadolinium MR images. An epidural abscess could present as a mass with hypointensity on T1-weighted imaging, hyperintensity on T2-weighted imaging, and rim enhancement. Inflammatory involvement of the vertebral body, involvement of other intervertebral discs, as well as coherent laboratory findings may provide important clues in case of epidural abscess. A hematoma usually has isointensity or hyperintensity on T1-weighted imaging, no enhancement, and an associated trauma history. Synovial cysts have a characteristic MR imaging signal intensity, and are related to the facet joint.

Because the MRI appearances of PEMLDF are not specific and are similar to those of other posterior epidural lesions, anamnesis and laboratory findings are critical to preoperative diagnosis. A definitive diagnosis can at times be made intraoperatively.^[14]^ Takano et al used discography and disco-CT to distinguish PEMLDF from other spinal pathologies, such as malignancy, spontaneous hematoma, or epidural abscess, and make a definite diagnosis of PEMLDF.^[14]^ They also pointed out that discography allows surgeons to distinguish the L3-L4 lumbar disc herniation from other segments and to understand the entire aspect of the PEMLDF.

Management of PEMLDF should follow the guidelines that apply in cases of ordinary disc herniation. However, approximately 50% of cases are emergencies, which make decision-making and treatment planning challenging.^[8]^ In most cases, surgery is the treatment of choice. Surgical management was carried out in 96% of the cases reviewed by Elshakawy et al, and in most cases the surgical intervention was satisfactory.^[4]^ Other authors have recommended that surgery should be performed without delay in patients with large sequestrated disc fragments and neurological deficits, and point out that this is particularly important in patients with CES.^[1]^ Laminectomy and decompression have been the surgical strategies predominantly used in cases of PEMLDF. Laminectomy ensures full exposure of the fragment and easier removal of the lesion, decreases the risk of incidental dural tear, minimizes the traction on the neural structures, and saves time, which is especially important in emergency situations.^[14]^ An extra discectomy may be called for, particularly when a rent is present in the posterior longitudinal ligament. Among the patients in our case series, the sequestrated disc fragment was removed either via laminectomy or hemi-laminectomy. (Table 1)

Fortunately, the prognosis of CES secondary to PEMLDF is considerably better than that of CES resulting from an anteriorly extruded disc fragment. According to the previously published reports, 53 (73.62%) patients made a total recovery, 3 (4.17%) had a subtotal recovery, 15 (20.83%) showed improvement, and 1 (1.38%) had unchanged clinical condition.^[1]^ Several authors have suggested that a fairly large amount of epidural fat at the posterior portion provides adequate space for cauda equina, allowing most PEMLDF patients to fully recover within weeks to months after surgery.^[1,5]^ Consistent with previous results, both of our patients with CES made a total recovery post-surgery.

## 4 Conclusion

PEMLDF is very rare. The condition may present with lower back pain, radicular compression, or CES. Preoperative diagnosis is difficult because of rim enhancement, which may mimic an intraspinal extradural tumor. The peripheral enhancement of the disc fragment on contrast-enhanced imaging studies is attributed to an inflammatory response with vascular granulation tissue around the disc mass. The patient history, the recognition of similar signal intensity between the mass and the intervertebral disc on MRI scans, and looking for peripheral rim enhancement are the keys to the correct diagnosis of PEMLDF.

## Author contributions

**Conceptualization:** Youngmin Oh.

**Funding acquisition:** Jongpil Eun.

**Visualization:** Youngmin Oh, Jongpil Eun.

**Writing – original draft:** Jongpil Eun, Youngmin Oh.

**Writing – review & editing:** Jongpil Eun, Youngmin Oh.
